# Structure-regulated enhanced Raman scattering on a semiconductor to study temperature-influenced enantioselective identification†

**DOI:** 10.1039/d4sc00855c

**Published:** 2024-04-18

**Authors:** Jing Xu, Junhan Li, Xuao Liu, Xu Hu, Hairihan Zhou, Zhida Gao, Jingwen Xu, Yan-Yan Song

**Affiliations:** a Department of Chemistry, College of Sciences, Northeastern University Shenyang 110819 China yysong@mail.neu.edu.cn xujingwen@mail.neu.edu.cn; b State Key Laboratory of Medicinal Chemistry and Molecular Diagnosis of the Ministry of Education, College of Chemistry & Materials Science, Hebei University Baoding 071002 China

## Abstract

Surface-enhanced Raman scattering (SERS) spectroscopy is an effective technique that can reveal molecular structure and molecular interaction details. Semiconductor-based SERS platforms exhibit multifaceted tunability and unique selectivity to target molecules as well as high spectral reproducibility. However, the detection sensitivity of semiconductors is impeded by inferior SERS enhancement. Herein, a surface and interference co-enhanced Raman scattering (SICERS) platform based on corrugated TiO_2_ nanotube arrays (c-TiO_2_ NTs) was developed, and the coupling of structural regulation and photo-induced charge transfer (PICT) effectively optimized the SERS performance of the semiconductor substrate. Due to the regularly oscillating optical properties of the c-TiO_2_ NTs, well-defined interference patterns were generated and the local electric field was significantly increased, which greatly promoted both the electromagnetic mechanism and PICT processes. The c-TiO_2_ NTs were subsequently applied as a highly sensitive SICERS substrate to investigate the mechanism of temperature influence on enantioselective identification. This identification process is related to the existence of temperature-sensitive hydrogen bonds and π–π interaction. This work demonstrates a simply prepared, low-cost, and sensitive SERS substrate that enables better investigation into molecular identification.

## Abstract 2

Combining structural regulation with photo induced charge transfer (PICT), a surface and interference co-enhanced Raman scattering (SICERS) platform was developed on a corrugated TiO_2_ nanotube arrays (c-TiO_2_ NTs).

## Graphical abstract

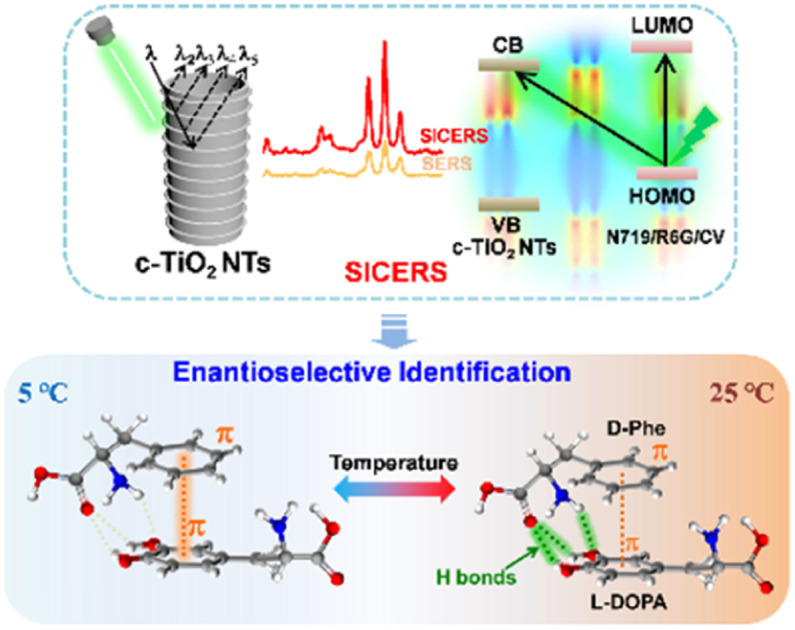

## Introduction

Surface-enhanced Raman scattering (SERS) is a powerful technique for trace analysis. SERS offers insights into the chemical structure and composition of molecules, enabling a wide range of potential applications across various fields ranging from nanostructure characterization to biochemical analysis.^1–3^ Conventional SERS substrates are based on the electromagnetic mechanism (EM),^4^ which utilizes the localized surface plasmon resonance (LSPR) effect of incident light excitation on a rough-surfaced metal to locally amplify an electromagnetic field.^5,6^ Typical EM strategies involve the construction of noble metal nanostructures with small gaps to generate “hotspots” and enhance the Raman scattering of nearby molecules. However, the signals of traditional EM-based SERS substrates significantly fluctuate due to the inherently non-uniform distribution of hotspots in the plasmonic nanostructures, and the poor selectivity of these substrates for target molecules usually results in complicated signal outputs. Another SERS mechanism is the chemical mechanism, which is derived from the efficient photo-induced charge transfer (PICT) that occurs between a substrate and molecules. PICT amplifies both the molecular polarizability tensor and Raman scattering cross-section.^7,8^ To date, the cost-effective fabrication, high spectral stability, and repeatability of semiconductors enable them competitive SERS substrates. Unfortunately, owing to their short-range charge transfer (CT) processes, the sensitivity of semiconductor-based SERS substrates is still much lower than that of noble metal-based SERS substrates.

Many strategies have been developed to enhance the performance of semiconductor-based SERS substrates.^9^ Among them, defect engineering is a well-established solution that can effectively activate the innate SERS activity of semiconductor-based substrates. Generally, defect engineering alters the band structure, surface properties, and densities of state of semiconductors. This is achieved by using complicated synthesis routes or post-treatment steps to introduce surface defects, which endow semiconductors with enhanced CT efficiency.^10^ Moreover, the structure of semiconductors is a crucial factor in enhancing the Raman signal. For example, multiple reflection processes in SERS substrates can lead to light interference, generating interference-enhanced Raman scattering (IERS). By altering the nanostructure, the optical properties of semiconductors such as light reflection, absorption, and interference can be regulated, which affects the electromagnetic field.^11,12^ Enhanced light absorption and LSPR at the nanostructure of semiconductors produce an enhanced interaction between different light routes and matter at optical hotspots, where the localized electromagnetic field is maximized. To date, few efforts have been made to integrate nanostructure-designed strategies and PICT processes to broaden the application prospects of semiconductor-based SERS substrates.

**Fig. 1:**
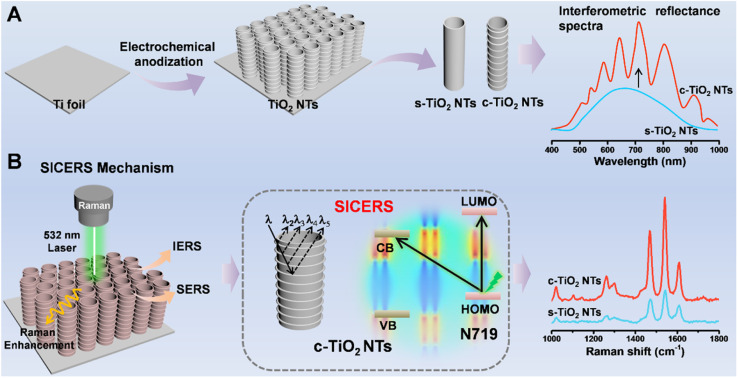
(A) Schematic illustration of the fabrication of c-TiO_2_ NTs SICERS substrate. (B) SICERS mechanism of the c-TiO_2_ NTs with significant signal enhancement.

TiO_2_ nanostructures are typical semiconductor nanomaterials that show advantages in terms of stability, economy, and biocompatibility. Among the various forms of TiO_2_, TiO_2_ nanotube arrays (TiO_2_ NTs) fabricated *via* electrochemical anodization have a regular geometric morphology, making them excellent candidates for use as SERS substrates.^13–16^ However, typical TiO_2_ NTs show poor SERS activity and are merely used as uniform substrates for plasmonic metal.^17^ In our previous work, we found that tailoring the water content of the electrolyte enabled the formation of TiO_2_ NTs with a corrugated surface capable of generating an interference signal.^18^ The interference of light is related to multiple reflection processes at material interfaces,^19,20^ and IERS effects lead to the generation of amplified Raman signals.^21–23^ Herein, by combining chemical enhancement and nanostructure regulation strategies, a substrate containing corrugated TiO_2_ NTs (c-TiO_2_ NTs) with optical interference properties was fabricated by a simple preparation process for use as a surface and interference co-enhanced Raman scattering (SICERS) platform with enhanced sensitivity and high molecular selectivity (Fig. 1). By regulating the nanostructure of the TiO_2_ NTs, a concentrated electric field region was generated within hotspots, which facilitated both the EM effect and the PICT efficiencies of the semiconductor-based SERS substrate. The ultrasensitive and selective c-TiO_2_ NTs substrate was subsequently employed to investigate the influence of temperature on enantioselective identification, and the mechanism of this process was clarified. Prussian blue (PB), which had an amplified and interference-free Raman signal, was formed *in situ* on the chiral c-TiO_2_ NTs substrate. This enabled the quantitative analysis of 3,4-dihydroxyphenylalanine (l/d-DOPA) enantiomers. The enantioselective identification mechanism was ascertained by investigating the temperature-sensitive hydrogen bonds and π–π interactions between the chiral environment and DOPA isomers.

## Results and discussion

### Fabrication and characterization of SICERS substrate

c-TiO_2_ NTs with good optical interference properties were prepared *via* electrochemical anodization. During the nanotube formation process, the oscillating optical properties of the nanotubes were determined by the anodic oxidation conditions. The water content of the electrolyte affected the growth rate and etching rate of the nanotubes (*i.e.*, the chemical dissolution rate).^24^ Bis(tetrabutylammonium) dihydrogen bis(isothiocyanate) bis(2,2′-bipyridyl-4,4′-dicarboxylate) ruthenium(ii) (N719) molecules were selected as Raman probes to optimize the interference pattern of the TiO_2_ NTs. The strongest SERS signal, corresponding to the optimal interference conditions, was generated on the c-TiO_2_ NTs with a length of 1.73 µm (Fig. S1–S3†). According to our previous work, corrugated nanotube sidewalls are conducive to generating optical interference, and the nanotube sidewall morphology can be regulated by controlling the electrolyte water content.^18^ Fig. S4 and S5† show that the TiO_2_ NTs grown in an electrolyte with low water content had smooth tube walls, while the walls of the TiO_2_ NTs grown in an electrolyte with high water content had corrugated tube walls. However, excess water in the electrolyte accelerated the etching speed of the fluorine-rich layer between the nanotubes, leading to deformed nanotubes with a spongy appearance. Thus, the optimum water content of the electrolyte was 15% (Fig. S6†).

### SICERS properties and mechanism of c-TiO_2_ NTs

**Fig. 2:**
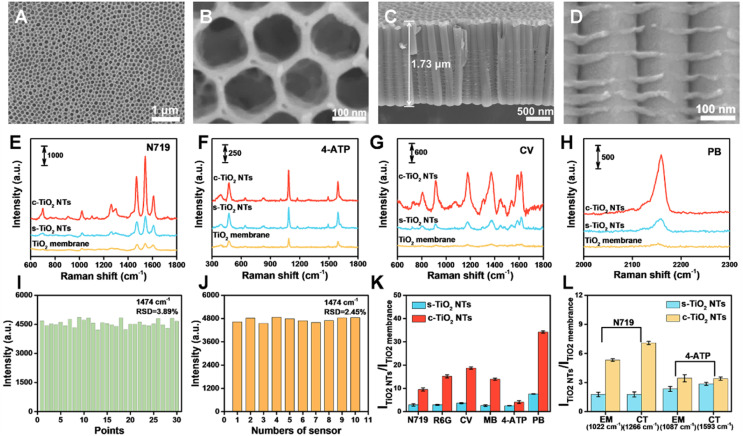
SEM images of (A and B) top view and (C and D) side view of c-TiO_2_ NTs prepared using an electrolyte with 15% water content. Raman measurement of (E) N719, (F) 4-ATP, (G) CV, and (H) PB on TiO_2_ membrane, s-TiO_2_ NTs, and c-TiO_2_ NTs. (I) Signal intensity of N719 at 1474 cm^−1^ collected from thirty random points on c-TiO_2_ NT-based substrates to demonstrate uniformity. (J) Signal intensity of N719 at 1474 cm^−1^ collected on ten different c-TiO_2_ NTs samples to demonstrate reproducibility. (K) Raman intensity ratios of N719, R6G, CV, MB, 4-ATP, and PB on the surfaces of the s-TiO_2_ NTs and c-TiO_2_ NTs to the TiO_2_ membrane. (L) Raman intensity ratios of N719 (at 1022 cm^−1^ and 1266 cm^−1^) and 4-ATP (at 1087 cm^−1^ and 1593 cm^−1^) on the surface of the s-TiO_2_ NTs and c-TiO_2_ NTs to the TiO_2_ membrane.

To gain the optimal Raman signal, the visible excitation wavelength dependence phenomena was investigated based on the Raman spectra of N719 under 532, 638, and 785 nm excitations (Fig. S7†). Under the 532 nm laser excitation, N719 exhibited the highest Raman signal intensity at the self-assembled monolayers level. Therefore, the 532 nm laser was used the excitation light in the following study. The SICERS properties of the c-TiO_2_ NTs and the effect of the TiO_2_ NTs tube wall morphology on the SERS signal were evaluated using a TiO_2_ nanoparticle-based membrane and TiO_2_ NTs with smooth tube walls (s-TiO_2_ NTs) as the control (Fig. S8†). The SEM characterizations in Fig. 2A–D show that the c-TiO_2_ NTs were composed of parallel and compact nanotubes with tube diameters of ∼155 nm. Numerous corrugations were observed on the tube walls of the c-TiO_2_ NTs, providing an optical interference phenomenon. Compared with the TiO_2_ nanoparticle-based membrane, the s-TiO_2_ NTs and c-TiO_2_ NTs showed stronger SERS signals after the adsorption of various probe molecules, including N719, 4-aminothiophene (4-ATP), crystal violet (CV), PB, rhodamine 6G (R6G), and methylene blue (MB) (Fig. 2E–H and S9†). The periodically ordered nanotubular structures of the s-TiO_2_ NTs and c-TiO_2_ NTs acted as photonic lattices to reflect light at specific frequencies, leading to enhanced Raman signals. Moreover, the periodically ordered nanostructures of the TiO_2_ NTs also provided uniform SERS signals, and the Raman spectra collected from 30 random points on a single sample showed a relative standard deviation (RSD) of 3.89% (Fig. 2I and S10A†). Meanwhile, the facile preparation of TiO_2_ NTs achieved a high reproducibility of 2.45% across ten different c-TiO_2_ NTs samples (Fig. 2J and S10B†). Interestingly, the SERS signals of most of the probe molecules were significantly enhanced by the c-TiO_2_ NTs substrate compared with the s-TiO_2_ NTs substrate. However, 4-ATP only showed a slight Raman enhancement on the c-TiO_2_ NTs substrate (Fig. 2K). A comparison of the Raman intensities of the N719 and 4-ATP peaks is shown in Fig. 2L. The non-symmetric mode of N719 at 1266 cm^−1^ (*ν*(C=N) (bpy) + *ν*(C–C) intern-ring (bpy) vibration mode) was significantly influenced by the CT process. The Raman shift of N719 at 1022 cm^−1^ was attributed to the symmetric vibration of the benzene ring, which was related to the LSPR contribution and was independent of the CT effect. Compared with the s-TiO_2_ NTs substrate, these N719 Raman peaks were significantly enhanced by the c-TiO_2_ NTs substrate. However, for 4-ATP, only a slight Raman enhancement was observed for the EM-affected peak at 1087 cm^−1^, while the CT influenced peak at 1593 cm^−1^ showed a negligible change in intensity. Based on the peak intensity of N719 at 1474 cm^−1^ on a silicon wafer and c-TiO_2_ NTs (Fig. S11†), the enhancement factors (EFs) of c-TiO_2_ NTs were estimated to be 6.1 × 10^4^. Although the EFs of c-TiO_2_ NTs were lower than some two-dimensional semiconducting materials like MXenes and graphitic carbon, c-TiO_2_ NTs exhibited highlights as good reproducibility, stability, and tailorability.

**Fig. 3:**
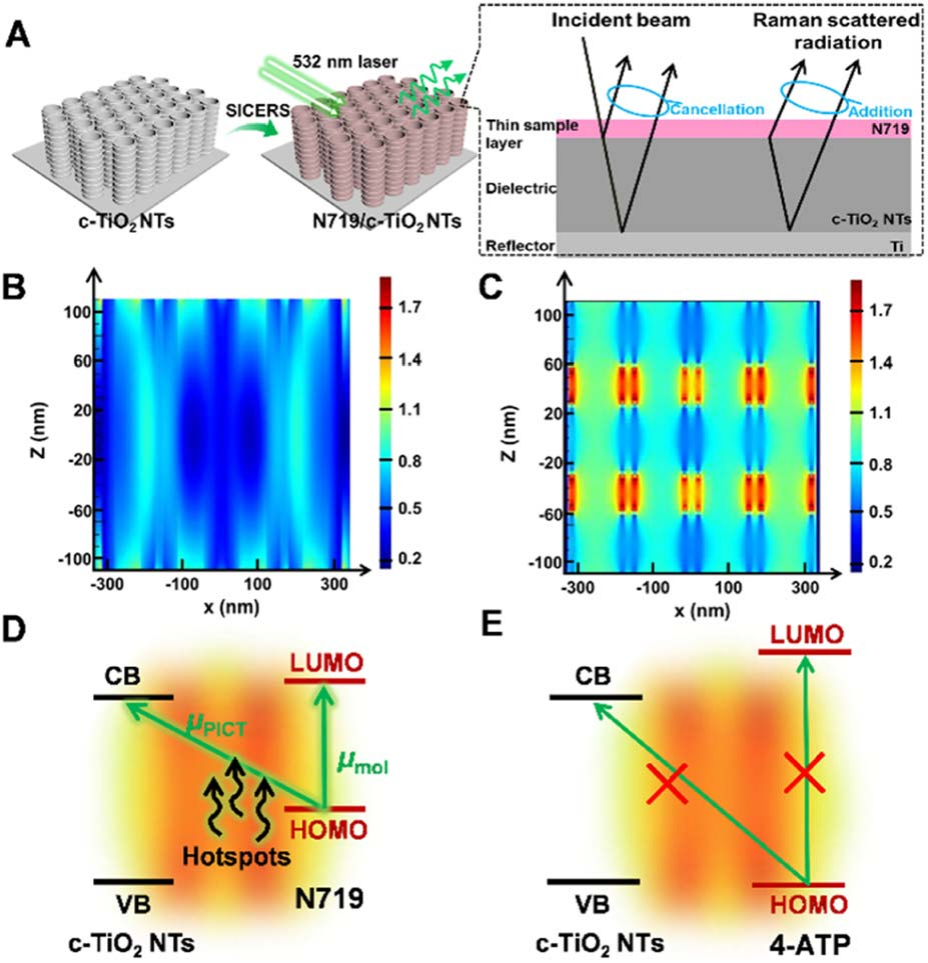
(A) Schematic diagram illustrating the SICERS effect of the c-TiO_2_ NTs and generation of Raman signal. Electromagnetic field enhancement images showing vertical sections of (B) s-TiO_2_ NTs and (C) c-TiO_2_ NTs. Energy level diagram and CT pathway of (D) N719 and (E) 4-ATP adsorbed on c-TiO_2_ NTs.

To investigate the enhanced SERS performance of the c-TiO_2_ NTs compared to the s-TiO_2_ NTs, the penetration process of the incident laser on the substrates was evaluated. According to the SERS mechanism, in Fig. 3A the collective excitation of electron gas or surface plasma of the conductor (in this case, the Ti substrate) was confined near the Ti/c-TiO_2_ NTs interface, and the light at the interface was significantly amplified under surface plasma excitation. As a result, both the incident radiation and reflected radiation at the Ti/c-TiO_2_ NTs interface were enhanced. According to the IERS mechanism, both the surface-enhanced reflected radiation and incident radiation contributed to the overall Raman scattering process in the IERS system, leading to a significant enhancement in SERS signal intensity. The finite difference time domain (FDTD) results in Fig. 3B and C show that the locally enhanced electric field of the c-TiO_2_ NTs presented a periodic distribution along the nanotubes (in the *z*-direction), and the field strength was significantly higher than that of the s-TiO_2_ NTs. Compared to the s-TiO_2_ NTs, stronger electromagnetic oscillation coupling between nanotubes was achieved in the c-TiO_2_ NTs, which enerated more hotspots in the substrate. Meanwhile, due to the periodic arrangement of the c-TiO_2_ NTs nanostructure, multiple light scattering between nanotube voids improved the light–matter interaction and increased the probability of Raman scattering.^25,26^ Therefore, a concentrated electric field region was formed within the c-TiO_2_ NTs nanostructure due to scattering and interference effects, which was also crucial for its light-harvesting properties.

The enhanced SERS performance of the c-TiO_2_ NTs for different molecules was further evaluated. The direct band gap (*E*_g_) of c-TiO_2_ NTs was determined to be ∼3.50 eV by Tauc plots analysis based on the UV-vis absorption spectrum (Fig. S12†). Both the non-symmetric bond and symmetric bands of N719 were significantly enhanced by the c-TiO_2_ NTs compared to the s-TiO_2_ NTs. As shown in Fig. 3D, under 532 nm (2.3 eV) light excitation, electrons were potentially transferred *via* two routes: (1) from the highest occupied molecular orbital (HOMO) of the prototype molecule N719 (−5.34 eV) to the conduction band (CB, −3.45 eV) of the c-TiO_2_ NTs; 2) from the HOMO to the lowest unoccupied molecular orbital (LUMO, −3.10 eV) of N719. In this case, the enhanced electric fields between the nanotubes in the c-TiO_2_ NTs substrate improved both the EM-based Raman scattering and facilitated effective PICT processes in the N719/c-TiO_2_ NTs system. Consequently, this system exhibits a significantly enhanced CT-based Raman signal compared to the N719/s-TiO_2_ NTs system. In contrast, the 4-ATP/TiO_2_ systems had unmatched band levels. Therefore, the CT process did not occur, and the c-TiO_2_ NTs only showed insignificant Raman enhancement compared to the s-TiO_2_ NTs (Fig. 3E). The other probe molecules that had matched energy levels with the c-TiO_2_ NTs also exhibited distinct enhanced SERS signals on the c-TiO_2_ NTs when compared to the s-TiO_2_ NTs (Fig. S13†). These results confirmed the vital impact of the hotspots generated by electromagnetic oscillation coupling between nanotubes in PICT processes.

### Construction of SICERS substrate for temperature-influenced enantioselective identification

**Fig. 4:**
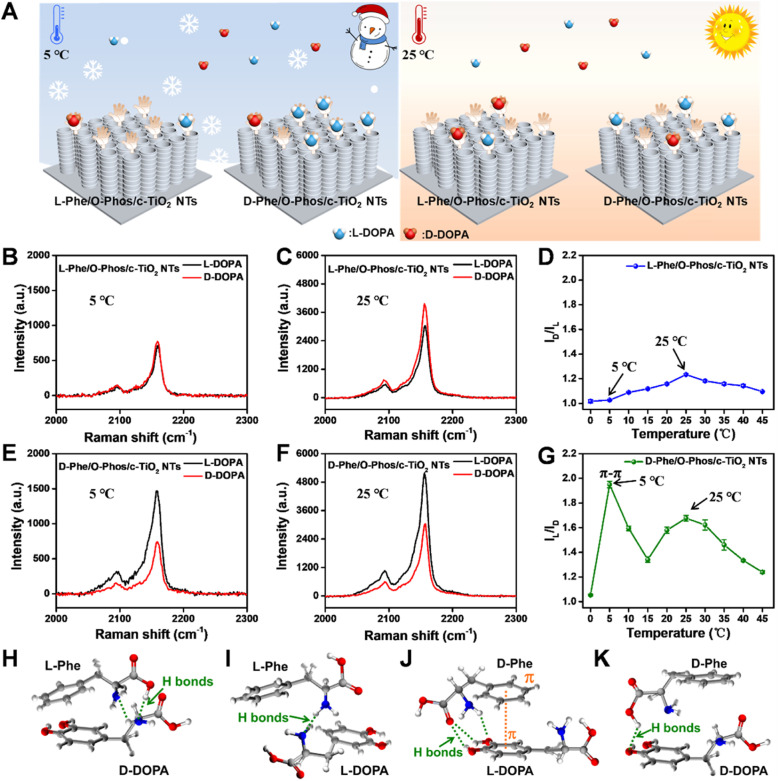
(A) Schematic illustrations showing the recognition of DOPA enantiomers by the l-Phe/O-Phos/c-TiO_2_ NTs and d-Phe/O-Phos/c-TiO_2_ NTs substrates at 5 °C and 25 °C. Raman spectra of PB after l/d-DOPA recognition by l-Phe/O-Phos/c-TiO_2_ NTs substrate at (B) 5 °C and (C) 25 °C. (D) Influence of temperature on the recognition of l/d-DOPA by the l-Phe/O-Phos/c-TiO_2_ NTs substrate. Raman spectra of PB after l/d-DOPA recognition by d-Phe/O-Phos/c-TiO_2_ NTs substrate at (E) 5 °C and (F) 25 °C. (G) Influence of temperature on the recognition of l/d-DOPA by the d-Phe/O-Phos/c-TiO_2_ NTs substrate. Energy-minimized dominant interaction models (ball-and-stick) of (H) l-Phe with d-DOPA, (I) l-Phe with l-DOPA, (J) d-Phe with l-DOPA, and (K) d-Phe with d-DOPA.

Identifying enantiomers and elucidating the recognition mechanism of an enantiomer identification strategy are significant challenges in bioanalysis because enantiomers have identical molecular formulas and chemical properties.^27–29^ Herein, the simple fabrication of c-TiO_2_ NTs SICERS substrates with high stability and reproducibility motivated us to develop a platform for investigating the mechanism of enantioselective identification. A chiral environment based on the c-TiO_2_ NTs was constructed to explore the effect of temperature on enantiomer (l/d-DOPA) identification (Fig. 4A and S14†). The characterization of this analysis platform (Fig. S15–S21†) demonstrated that the prepared l/d-Phe/O-Phos/c-TiO_2_ NTs SICERS substrates were constructed successfully with homochirality. Fig. S22† shows the construction of the chiral recognition signal amplification sensing platform. After l/d-DOPA identification, an Fe^3+^–DOPA complex was formed *in situ* through chelation between Fe^3+^ and the phenol hydroxyl group of DOPA.^15^ Fe^3+^ subsequently reacted with [Fe(CN)_6_]^4–^ to form PB nanocrystals. The selective recognition of DOPA enantiomers on the chiral SICERS substrate was determined by comparing the peak intensity of PB at 2158 cm^−1^. Considering that the DOPA molecules tend to self-polymerize in a basic solution,^30,31^ an acidic environment was thus chosen to ensure the DOPA monomer to be recognized on the substrate. To obtain the apparent recognition results, the reaction conditions were optimized as follows: the chiral recognition time was 60 min, the Fe^3+^ concentration was 0.5 mM, and the Fe^3+^ chelation time was 30 min (Fig. S23–S25†). To determine the influence of electrostatically adsorbed Fe^3+^ ions on DOPA sensing, we also explored the PB generation ability induced by the adsorbed Fe^3+^ ions on c-TiO_2_ NTs, O-Phos/c-TiO_2_ NTs, and l-Phe/O-Phos/c-TiO_2_ NTs (Fig. S26†). Apparently, very weak PB signals were detected on these samples, indicative the effective PB-growth strategy by using the recognized l/d-DOPA.

Previous studies have shown that the hydrogen bonds between the l/d-Phe and l/d-DOPA is crucial for the chiral recognition process, and the strength of this hydrogen bond is closely related to temperature.^32^ As predicted, chiral substrates exhibited different chiral recognition phenomena at different recognition temperatures (Fig. 4B–D). The PB Raman signal intensities obtained for the same concentrations of l/d-DOPA on the l-Phe/O-Phos/c-TiO_2_ NTs substrate at 5 °C significantly overlapped, suggesting that the target enantiomer was not able to be distinguished using the homochiral substrate at a low temperature. At 25 °C, the PB Raman intensity generated after d-DOPA identification by the l-Phe/O-Phos/c-TiO_2_ NTs substrate was higher than that of l-DOPA. For the d-Phe/O-Phos/c-TiO_2_ NTs substrate (Fig. 4E–G), the PB Raman intensities after recognizing l-DOPA at both 5 °C and 25 °C were significantly higher than that of d-DOPA. To understand the chiral recognition behavior of the substrates under different temperatures, chiral recognition based on l-Phe and d-Phe was examined at a range of temperatures from 0 to 45 °C (Fig. 4D and G). The results indicated that chiral selectivity for the DOPA enantiomer depended on the experimental temperature. The selectivity of the l-Phe/O-Phos/c-TiO_2_ NTs chiral sensor for d-DOPA increased with increasing temperature, and this chiral sensor exhibited the highest selectivity for d-DOPA, especially at 25 °C. Meanwhile, the d-Phe/O-Phos/c-TiO_2_ NTs chiral sensor showed high selectivity at both 5 °C and 25 °C. For other chiral molecules containing phenolic hydroxyl groups (such as l/d-adrenaline), the temperature also affected the chiral recognition process (Fig. S27†).

The interactions between the l/d-Phe and DOPA enantiomers were then investigated, as schematically shown in Fig. 4H–K. Both DOPA enantiomers interacted with l/d-Phe and formed hydrogen bonds. For l-Phe/O-Phos/c-TiO_2_ NTs, the interaction between d-DOPA and l-Phe involved the formation of two hydrogen bonds, while the interaction between l-DOPA and l-Phe formed one hydrogen bond. While for d-Phe/O-Phos/c-TiO_2_ NTs, l-DOPA bonded to d-Phe through three hydrogen bonds and π–π interaction, and d-Phe bonded to d-DOPA *via* only one hydrogen bond. Therefore, the temperature-influenced chiral recognition process was essentially decided by the different binding modes between the l/d-Phe and DOPA enantiomers. Low temperatures are favorable for π–π interaction, but molecular motion is limited at low temperatures. However, hydrogen bonds are significantly inhibited due to the limited molecular motion at low temperatures.^33,34^ Thus, with increasing temperature, the hydrogen bonds became more prominent, which enhanced the d-DOPA selectivity on the l-Phe/O-Phos/c-TiO_2_ NTs substrate (Fig. 4D). The d-Phe/O-Phos/c-TiO_2_ NTs substrate exhibited higher selectivity for l-DOPA at low temperatures, especially at 5 °C (Fig. 4G). This was attributed to the stable π–π interactions between d-Phe and l-DOPA at low temperatures. Molecular motion accelerates with increasing temperature due to the weakened intramolecular interactions. Thus, at 25 °C, the formation of hydrogen bonds between d-Phe and l-DOPA was enhanced. However, excessively high temperatures (30–45 °C) broke the stable hydrogen bonds between the l/d-Phe and DOPA enantiomers, resulting in a subsequent decline in recognition efficiency.

The quantitative analysis capability of l-Phe/O-Phos/c-TiO_2_ NTs was investigated (Fig. S28†). The PB Raman signal recorded on the SICERS platform exhibited good linear relationships in the l/d-DOPA concentration ranging from 10^−10^ to 10^−5^ M with the limit of detection (LOD, defined as the mean of blank +3*σ*, *σ* is the standard deviation of blank) of 1.3× 10^−11^ M for l-DOPA and 1.0 × 10^−11^ M for d-DOPA. The X-ray diffraction (XRD) patterns and SEM images also provided the solid evidence for the of PB formation on the surface of c-TiO_2_ NTs by the DOPA enantiomer recognition process (Fig. S29 and S30†). In addition, the selectivity of the SICERS platform was further evaluated by discriminating other enantiomers, including l/d-tryptophan (l/d-Trp), l/d-carnitine, l/d-tyrosine (l/d-Tyr), l/d-glutamic acid (l/d-Glu), and l/d-cysteine (l/d-Cys) (Fig. S31†). Owing to no phenolic hydroxyl groups in the structures of these enantiomers, Fe^3+^ ions cannot be trapped, and thus no obvious PB signal was recorded. The above results confirm the excellent quantitative ability of the as-proposed SICERS platform for DOPA enantiomers.

## Experimental

### Preparation of TiO_2_ NTs

Ti foils (15 mm × 15 mm × 0.1 mm) were sequentially cleaned by ultrasonication in isopropanol, ethanol, and deionized water, then dried under a flow of N_2_. The c-TiO_2_ NTs with optical interference properties were fabricated by electrochemical anodization.^18,24^ Briefly, each Ti foil was anodized at 60 V for 40 min in an electrolyte containing 0.5 wt% NH_4_F and varying water content to prepare the c-TiO_2_ NTs.

### Preparation of l/d-Phe/O-Phos/c-TiO_2_ NTs

The as-formed c-TiO_2_ NTs were first functionalized with O-Phos by immersion in a 10 mM O-Phos aqueous solution at 4 °C for 12 h. Subsequently, chiral recognition molecules (l/d-Phe) were introduced onto the prepared O-Phos/c-TiO_2_ NTs using a classic *N*-(3-dimethylaminopropyl)-nethylcarbodiimide hydrochloride (EDC)/*N*-hydroxysulfosuccinimide (NHS) coupling reaction. To link l/d-Phe onto the O-Phos/c-TiO_2_ NTs, 1 mM l/d-Phe was dissolved in deionized water containing 10 mg mL^−1^ EDC and 5 mg mL^−1^ NHS. This mixture was reacted for 30 min to activate the carboxyl group of l/d-Phe. The O-Phos/c-TiO_2_ NTs were soaked in this solution for 12 h at room temperature to obtain the l/d-Phe/O-Phos/c-TiO_2_ NTs.

### Temperature induced chiral recognition for l/d-DOPA

Firstly, the as-prepared l/d-Phe/O-Phos/c-TiO_2_ NTs were immersed in l/d-DOPA (pH 5.5) aqueous solution at different temperatures for 60 min. After immersion, the substrates were cleaned with deionized water three times to remove uncombined l/d-DOPA molecules. The cleaned samples were then incubated in 0.5 mM FeCl_3_ aqueous solution for 30 min to fix Fe^3+^. Subsequently, PB was formed by immersing the samples in a 0.5 mM K_4_[Fe(CN)_6_] aqueous solution at room temperature for 30 min.

### Raman measurement

The c-TiO_2_ NTs were immersed in a 1 mM ethanol solution containing N719 and reacted at 70 °C for 12 h. Raman spectra were recorded using a 532 nm laser (10% power) equipped with a 50× long-distance objective and a spot size of 1 µm. The data acquisition time was 10 s, the confocal hole size was 500 mm, and the slit aperture size was 200 mm. The spectrometer was calibrated using the Raman signal from a silicon wafer at 520.7 cm^−1^. For each measurement, Raman spectra were collected from five different locations to determine the average signal strength.

### Calculation of the electromagnetic surface enhancement in TiO_2_ NTs geometries

The field intensity distribution of the c-TiO_2_ NTs and s-TiO_2_ NTs was calculated by FDTD. The experimental parameters for the calculations were obtained from the SEM images of the c-TiO_2_ NTs and s-TiO_2_ NTs. In the FDTD simulations, four TiO_2_ NTs were used as the model, 0.3 nm was used as a unit, and a 532 nm laser was used to irradiate the model along the *z* and *y* axes.

## Conclusions

In summary, a c-TiO_2_ NTs substrate with a facilely tailorable nanostructure was proposed in this work as a SICERS platform due to its enhanced Raman scattering properties. This platform was employed to investigate the influence of temperature on enantioselective identification and the mechanism of this influence. By regulating the TiO_2_ NTs nanostructure, an enhanced electric field with interference properties was generated in the c-TiO_2_ NTs, facilitating both the EM and PICT processes in the semiconductor-based SERS substrate. A signal amplification strategy involving the *in situ* formation of PB was employed to determine that temperature-sensitive hydrogen bonds and π–π interactions between the chiral environment of the substrate and isomers were the key to enantioselective recognition. This work offers new opportunities for improving the sensitivity of semiconductor-based SERS substrates with simple methods and is expected to be helpful for biochemical analysis, catalyst monitoring, and material determination.

## Data availability

All the data supporting this article have been included in the main text and the ESI.†

## Author contributions

Y.-Y. Song and J. W. Xu conceived the concept and directed the project. J. Xu performed experiments, analyzed data, and wrote the manuscript. J. H. Li, X. Hu, H. R. H. Zhou performed the experiments. X. A. Liu carried out the theoretical study. Z. D. Gao collected and analyzed the data. All the authors discussed the results and assisted during the manuscript preparation.

## Conflicts of interest

There are no conflicts to declare.

## Supplementary Material

SC-015-D4SC00855C-s001
