# Supplementary material for: Structure-regulated enhanced Raman scattering on a semiconductor to study temperature-influenced enantioselective identification
Source: Chem Sci. 2024 Apr 18;15(19):7308–15. doi: 10.1039/d4sc00855c (PMC11095390; doi:10.1039/d4sc00855c)
Supplement: SC-015-D4SC00855C-s001 [file SC-015-D4SC00855C-s001.pdf]

Electronic Supplementary Material (ESI) for Chemical Science.
This journal is © The Royal Society of Chemistry 2024

Supporting Information

Structure-regulated enhanced Raman scattering on a semiconductor to study temperature-influenced enantioselective identification

Jing Xu,[a,b] Junhan Li,[a] Xuao Liu,[a] Xu Hu,[a] Hairihan Zhou,[a] Zhida Gao,[a] Jingwen Xu*[a] and Yan-Yan Song*[a]

[a]Department of Chemistry, College of Sciences, Northeastern University, Shenyang 110819, China

[b]State Key Laboratory of Medicinal Chemistry and Molecular Diagnosis of the Ministry of Education, College of Chemistry & Materials Science, Hebei University, Baoding 071002, China

Corresponding Authors

* Yan-Yan Song, E-mail: yysong@mail.neu.edu.cn

* Jingwen Xu, E-mail: xujingwen@mail.neu.edu.cn

EXPERIMENTAL SECTION

Materials and reagents. Ti sheets (0.1 mm thickness, 99.6% purity) were purchased from Baosheng Hardware (Bao ji). O-phosphorylethanolamine ($C_2H_8NO_4P$), phenylalanine ($C_9H_{11}NO_2$), tryptophan ($C_{11}H_{12}N_2O_2$), carnitine ($C_7H_{15}NO_3$), tyrosine ($C_9H_{11}NO_3$), cysteine ($C_3H_7NO_2S$), potassium ferrocyanide trihydrate ($K_4FeC_6N_6 \cdot 3H_2O$), iron chloride hexahydrate ($FeCl_3 \cdot 6H_2O$), and bis(tetrabutylammonium) dihydrogen bis(isothiocyanate) bis(2,2'-bipyridyl-4,4'-dicarboxylate) ruthenium(II) ($C_{58}H_{84}N_8O_8RuS_2$) were purchased from Aladdin (Shanghai, China). N-Hydroxysuccinimide (NHS) and N-(3-Dimethylaminopropyl)-3-ethylcarbodiimide hydrochloride (EDC) were obtained from Sigma-Aldrich (St. Louis, USA). Ammonium fluoride (NH_4F), ethylene glycol, glutamic acid ($C_5H_9NO_4$), isopropanol (C_3H_8O), and ethanol (C_2H_5OH) were purchased from Sinopharm Chemical Reagent Co. Ltd. and used as received without further purification. All aqueous solutions were prepared using Millipore Milli-Q water with a resistivity of 18.0 MΩ·cm.

Apparatus and characterization. Interference reflectance spectra of c-TiO_2 NTs were collected by using an USB 2000+ fiber optic spectrometer (Ocean Optics, USA) coupled with a bifurcated fiber optic cable (Ocean Optics, USA). Raman measurements were conducted using a Raman microscopy spectrometer (LabRAM HR, HORIBA Scientific, France). Morphological characterization was carried out using a field-emission scanning electron microscope (SEM, Hitachi SU8000, Japan). Crystal structures were identified by XRD acquired using an X'Pert XRD spectrometer (Philips, USA) using a CuKα X-ray source. Zeta potentials were measured on a Zetasizer Nano ZS90 analyzer (Malvern, USA). The chiral optical properties were recorded by circular dichroism (CD) spectra using a MOS-450 CD Spectrometer (Bio-Logic, France). X-ray photoelectron spectra (XPS) were recorded on a Perkin-Elmer Physical Electronics 5600 spectrometer using AlKα radiation at 13 kV as excitation source. Fourier transform infrared (FTIR) spectroscopy was performed using a Nicolet 6700 instrument (Thermo Fisher, USA).

Calculation of EFs. The EFs of c-TiO_2 NTs were determined according to the following equation.[1,2]

$$\text{EFs} = \left(\frac{I_{SICERS}}{I_{bulk}}\right)\left(\frac{N_{bulk}}{N_{SICERS}}\right) \quad \text{(S1)}$$

$$\text{N} = \text{CV}N_A S_{sub} \quad \text{(S2)}$$

where I_{SICERS} and I_{bulk} are the intensities of the selected Raman peak of N719 on the SICERS substrate and pristine silicon wafer, N_{bulk} and N_{SICERS} are the average number of N719 molecules in the scattering area used for Raman and SICERS measurements, respectively. The spectrum of N719 (1 mM) on the pristine silicon wafer were used as the normal Raman reference. The number of probe molecules within the scattering area was estimated using Equation 2 based on the assumption that the probe molecules were uniformly distributed on the substrates. C represents the molar concentration of the N719 solution, V is the volume of the droplet, N_A is Avogadro's constant, and S_{sub} is the effective area of the substrate.

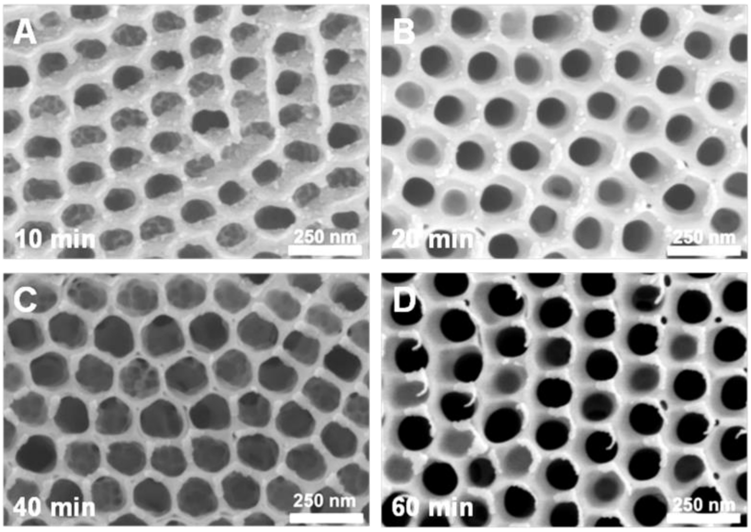

Fig. S1. SEM images of top view of TiO_2 NTs prepared with different anodization periods: (A) 10 min, (B) 20 min, (C) 40 min, and (D) 60 min.

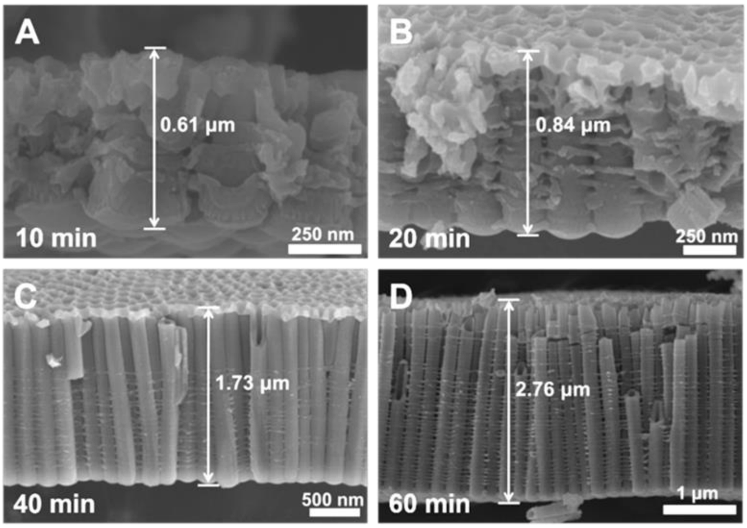

Fig. S2. SEM images of side view of TiO_2 NTs prepared with different anodization periods: (A) 10 min, (B) 20 min, (C) 40 min, and (D) 60 min.

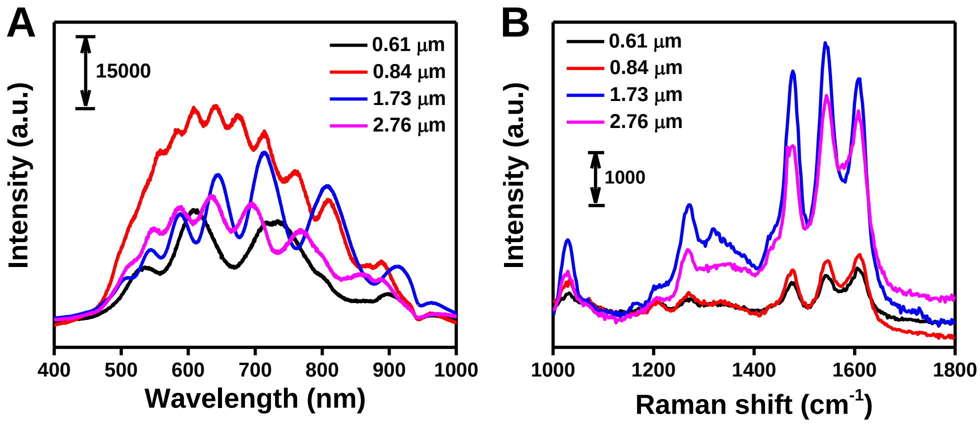

Fig. S3. (A) Interferometric reflectance spectra of TiO_2 NTs with different lengths. (B) Raman spectra of N719 adsorbed on TiO_2 NTs with different lengths.

Fig. S1 and S2 show the SEM images of TiO_2 NTs prepared with different anodization times. The nanotube length and the number of interference corrugations were mainly controlled by the anodization time. The nanotube length influenced the propagation path of incident light inside the nanotube arrays, which in turn affected the interference effect of the substrate. An appropriate nanotube length was conducive to the generation of interference peaks with regular interference corrugations. Fig. S3A and B display the interferometric reflectance spectra and Raman spectra of TiO_2 NTs with varying lengths.

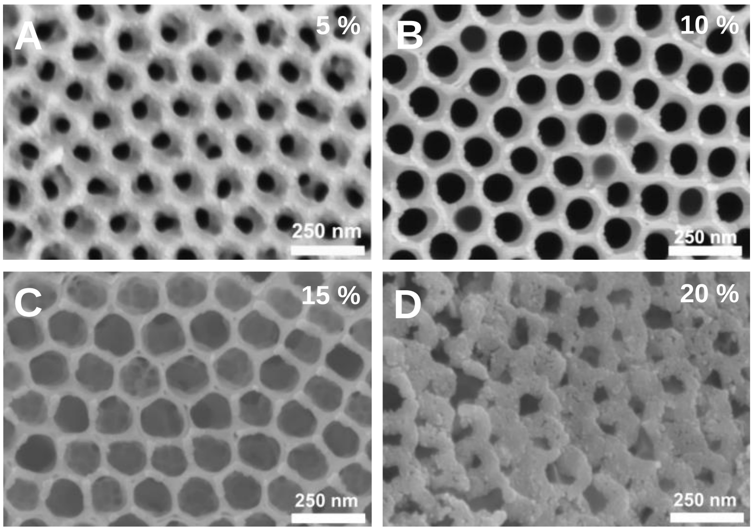

Fig. S4. SEM images of top view of TiO_2 NTs prepared using electrolytes with different water concentrations: (A) 5%, (B) 10%, (C) 15%, and (D) 20%.

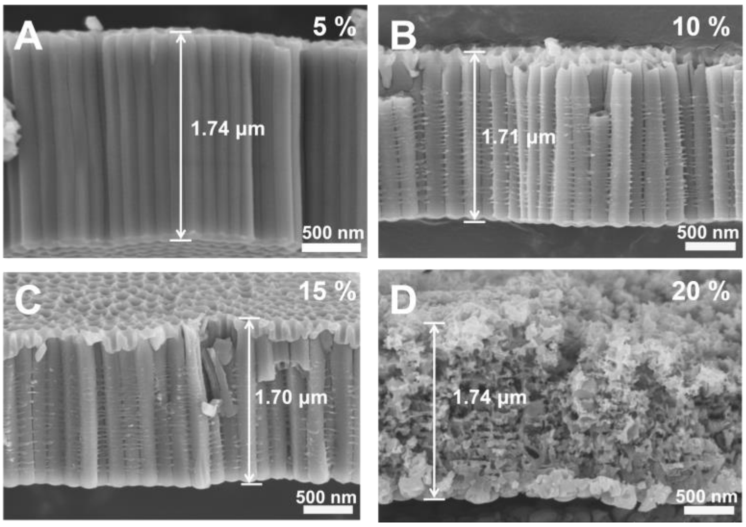

Fig. S5. SEM images of side view of TiO_2 NTs prepared using electrolytes with different water concentrations: (A) 5%, (B) 10%, (C) 15%, and (D) 20%.

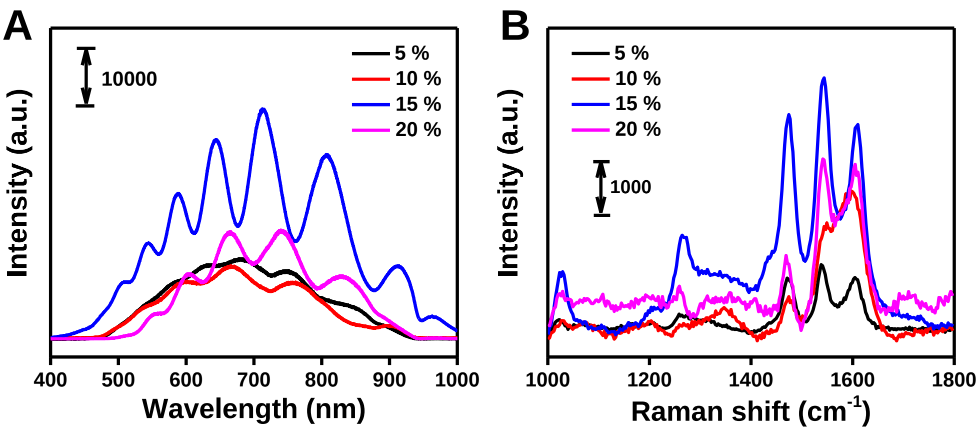

Fig. S6. (A) Interferometric reflectance spectra of TiO_2 NTs prepared using electrolytes with different water concentrations. (B) Raman spectra of N719 adsorbed on TiO_2 NTs prepared using electrolytes with different water concentrations.

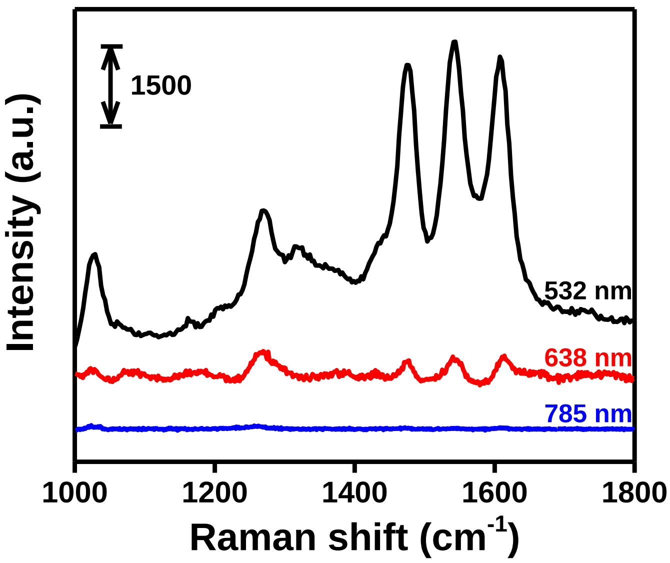

Fig. S7. Raman spectra of N719/c-TiO_2 NTs at 532, 638, and 785 nm excitation wavelengths.

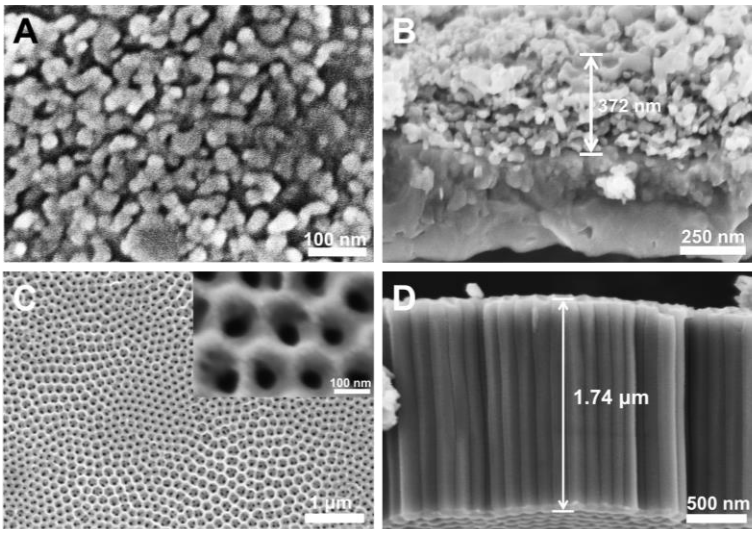

Fig. S8. SEM images of (A) top view and (B) side view of TiO_2 nanoparticles-based membranes. SEM images of (C) top view and (D) side view of s-TiO_2 NTs.

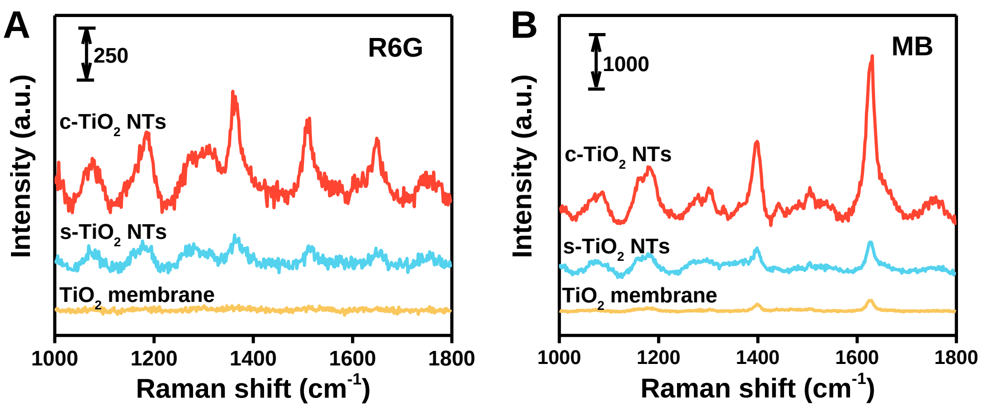

Fig. S9. Raman measurement of (A) R6G and (B) MB on TiO_2 membrane, s-TiO_2 NTs, and c-TiO_2 NTs.

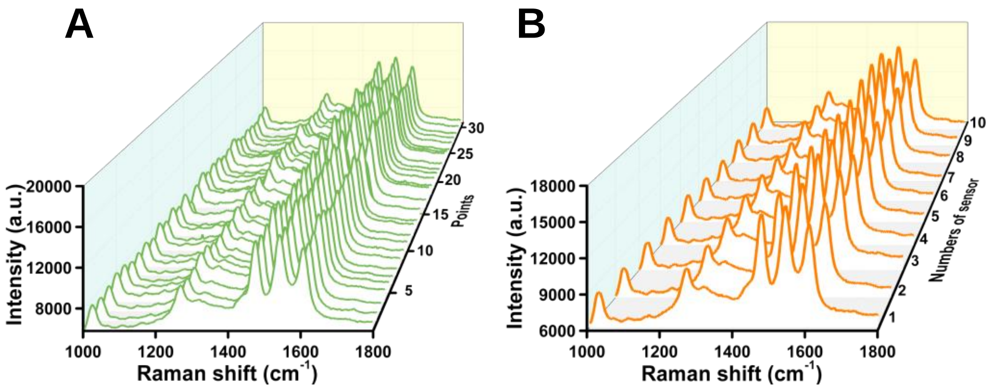

Fig. S10. (A) Raman spectra of N719 molecules collected on the thirty random points of c-TiO_2 NTs and (B) Raman spectra of N719 acquired on ten pieces of c-TiO_2 NTs.

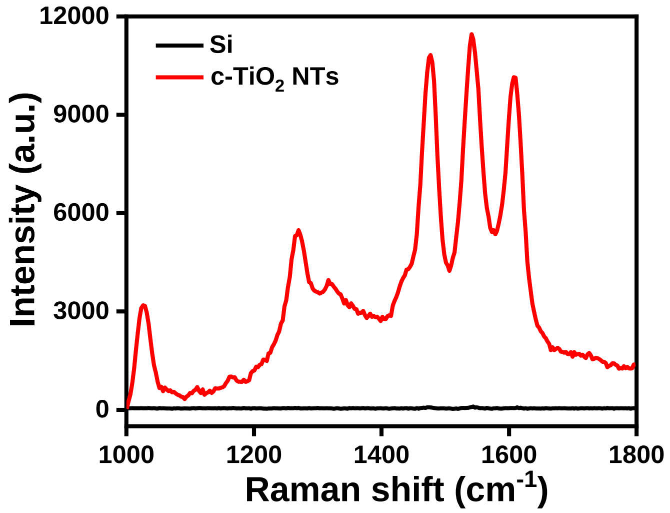

Fig. S11. Raman spectra of N719 adsorbed silicon wafer and c-TiO_2 NTs.

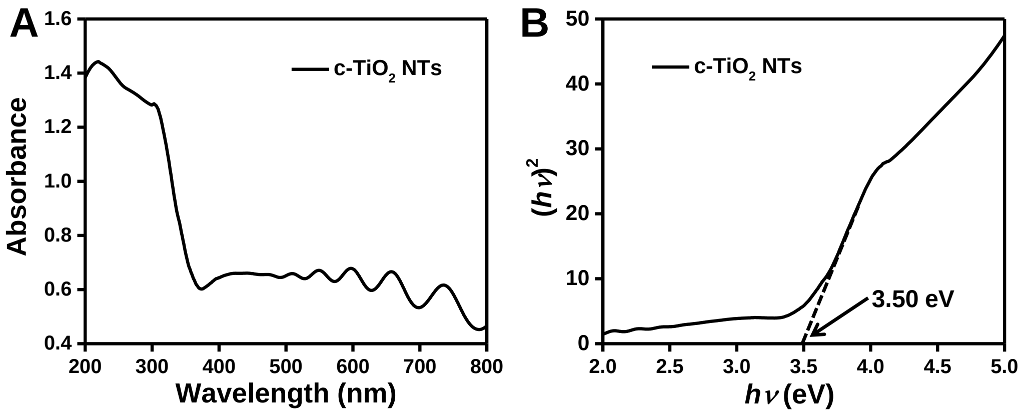

Fig. S12. (A) UV-vis absorption spectrum and (B) Tauc plots of c-TiO_2 NTs.

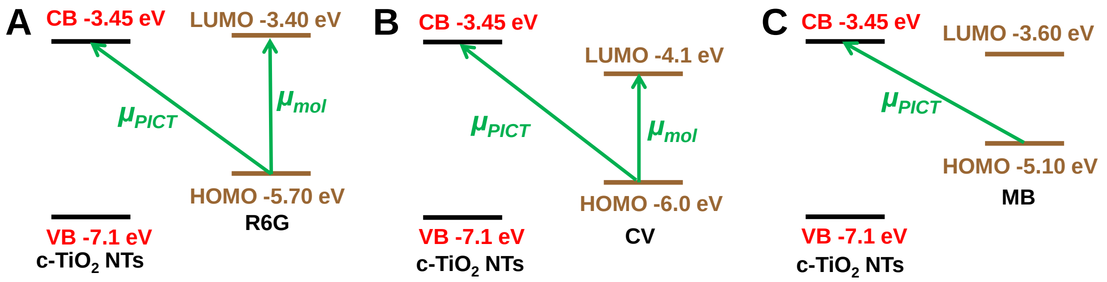

Fig. S13. Energy level diagram and CT pathway of (A) R6G, (B) CV, and (C) MB adsorbed on c-TiO_2 NTs.

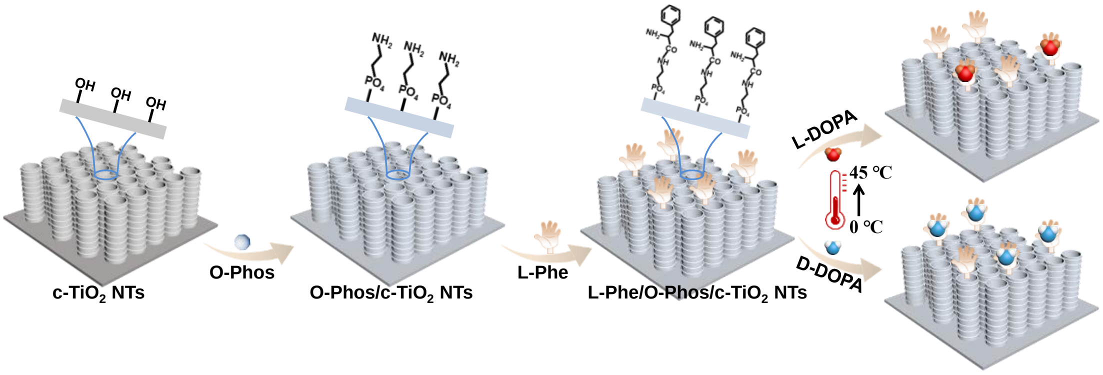

Fig. S14. Schematic illustrations showing the construction of chiral SICERS substrate for sensing DOPA enantiomers.

The as-prepared c-TiO_2 NTs had an abundance of surface hydroxyl groups (Ti-OH), which enabled the easy self-assembly of an O-Phos monolayer on the TiO_2 surface through its affinity with Ti-OH.[3] L/D-Phe was then bound to O-Phos through amide bonds to construct a homochiral environment.

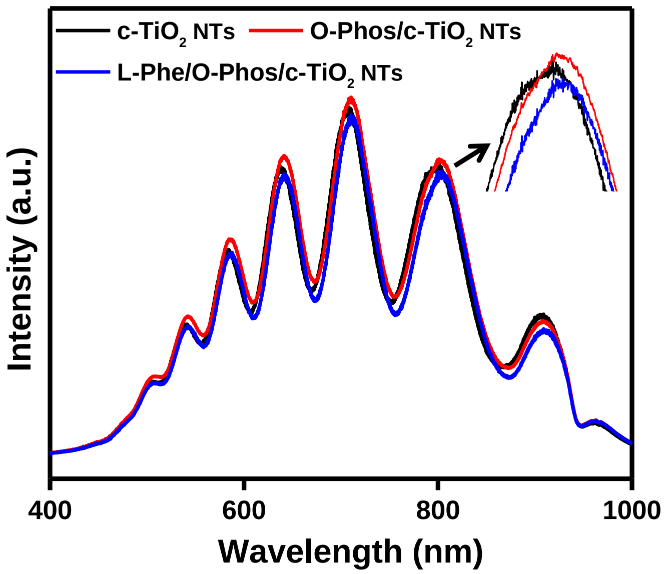

Fig. S15. Interferometric reflectance spectra of of c-TiO_2 NTs, O-Phos/c-TiO_2 NTs, and L-Phe/O-Phos/c-TiO_2 NTs.

Modifying the molecule on the substrate surface caused a change in the effective refractive index of the medium.[4] Thus, the interference peaks of the interferometric reflectance spectra were red-shifted after the O-Phos and L-Phe modifications.

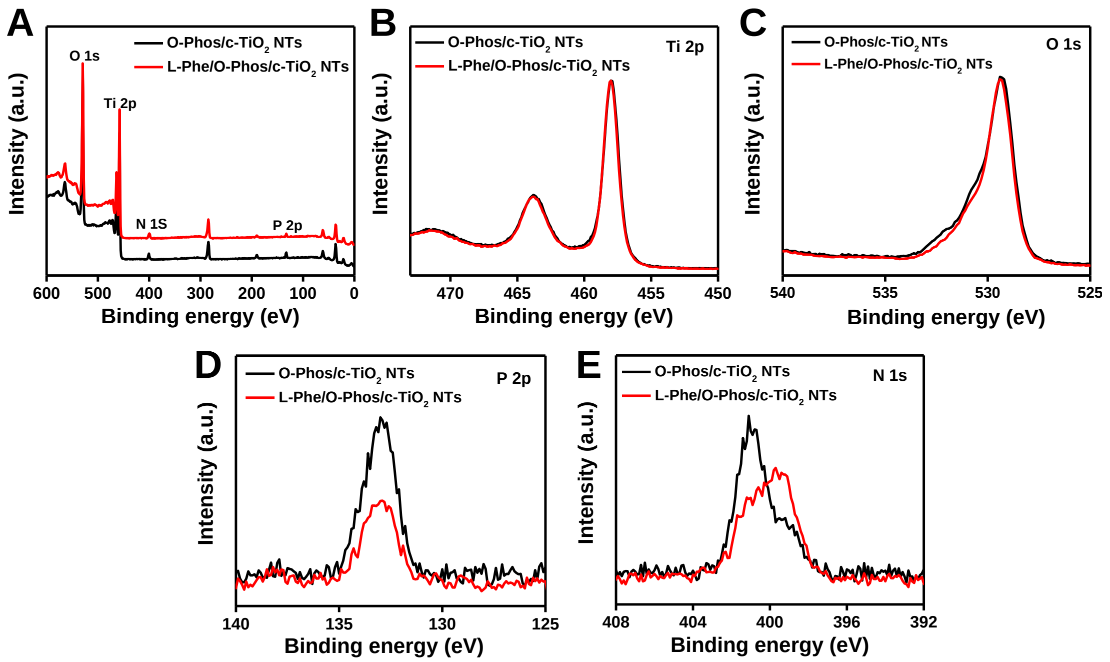

Fig. S16. (A) XPS survey spectra, (B) Ti 2p, (C) O 1s, (D) P 2p, and (E) N 1s spectra of O-Phos/c-TiO_2 NTs before and after L-Phe modification.

XPS analysis revealed that the obtained sample (L-Phe/O-Phos/c-TiO_2 NTs) consisted of Ti, O, P, and N elements without other impurities. The appearance of P 2p and N 1s signals demonstrated the successful modification of the c-TiO_2 NTs with O-Phos and L-Phe, respectively.

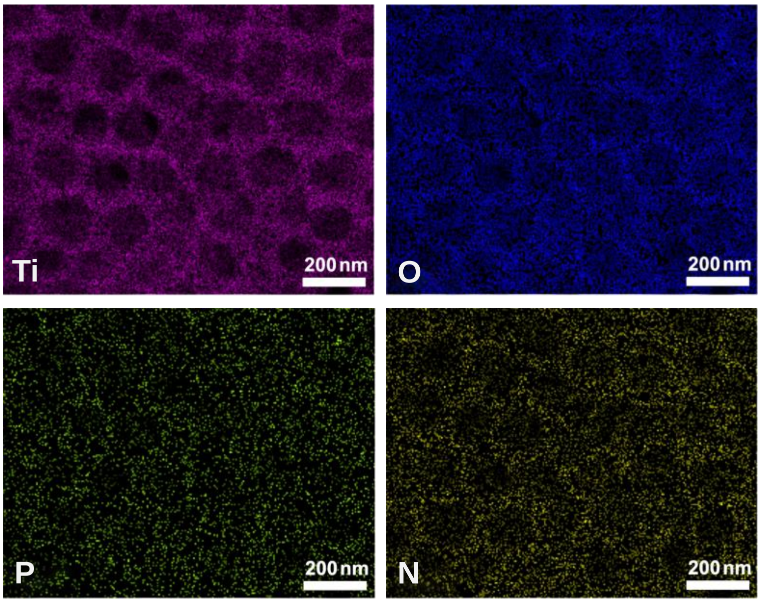

Fig. S17. The energy-dispersive X-ray spectroscopy (EDS) mapping images of L-Phe/O-Phos/c-TiO_2 NTs.

EDS mapping analysis further confirmed the uniform distribution of P and N elements on the surface of the c-TiO_2 NTs.

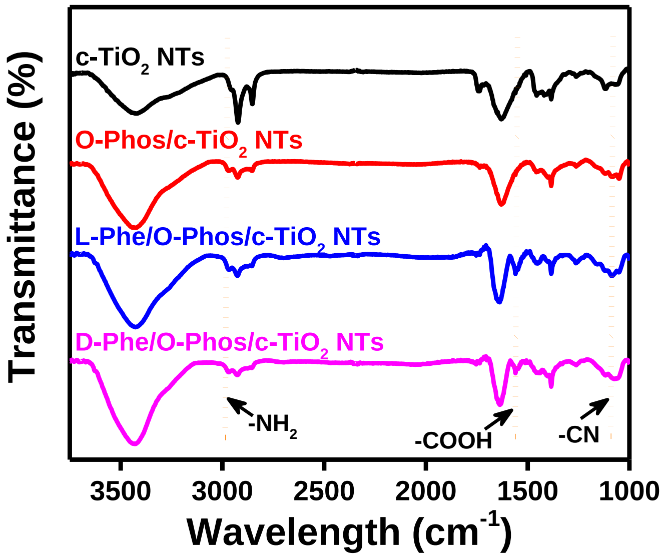

Fig. S18. FTIR spectra of c-TiO_2 NTs, O-Phos/c-TiO_2 NTs, L-Phe/O-Phos/c-TiO_2 NTs, and D-Phe/O-Phos/c-TiO_2 NTs.

The FTIR spectra of the L/D-Phe/O-Phos/c-TiO_2 NTs samples at each modification stage verified the successful modification of the O-Phos/c-TiO_2 NTs with L/D-Phe. The characteristic peaks at ~1087 cm^{-1} (-CN), 1562 cm^{-1} (-CO), and 2971 cm^{-1} (-NH_2) in the FTIR spectra were attributed to O-Phos and L/D-Phe.

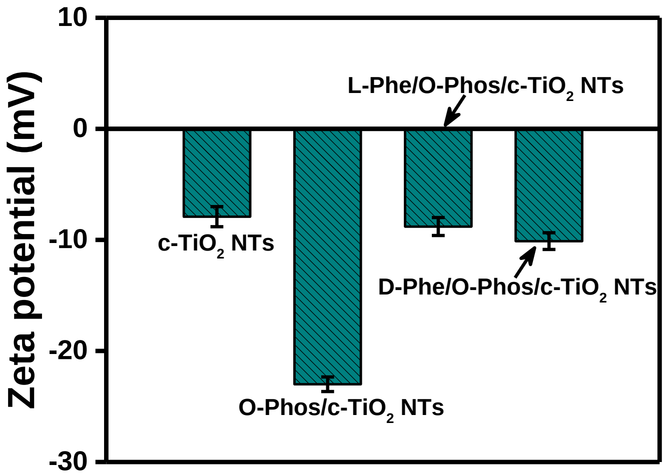

Fig. S19. Zeta potential of c-TiO_2 NTs, O-Phos/c-TiO_2 NTs, L-Phe/O-Phos/c-TiO_2 NTs, and D-Phe/O-Phos/c-TiO_2 NTs.

The substrate had different zeta potentials after O-Phos and L/D-Phe modification due to the changes in the functional groups on the nanotube surface.

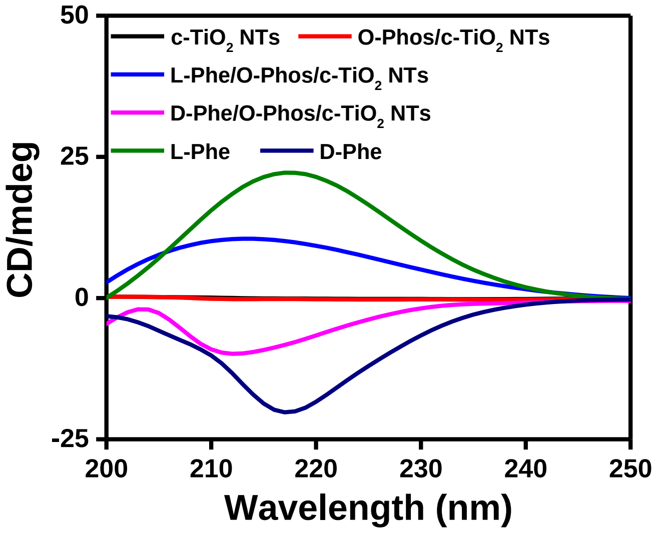

Fig. S20. CD spectra of c-TiO_2 NTs, O-Phos/c-TiO_2 NTs, L-Phe/O-Phos/c-TiO_2 NTs, D-Phe/O-Phos/c-TiO_2 NTs, and L/D-Phe.

To determine the homochiral characteristics of the prepared SICERS substrates, the CD spectra of the c-TiO_2 NTs were obtained after each stage of modification. An adsorption peak at 217 nm in the CD spectra of the L/D-Phe/O-Phos/c-TiO_2 NTs, which was consistent with the CD peak of pure L/D-Phe, indicating that the constructed L/D-Phe/O-Phos/c-TiO_2 NTs SICERS substrate was homochiral.

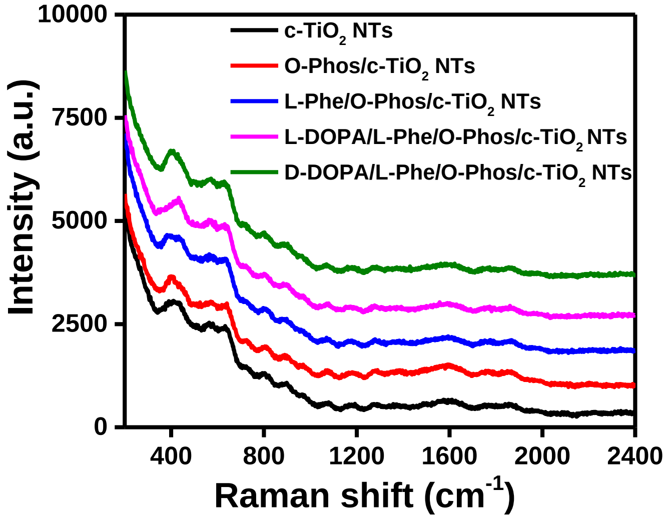

Fig. S21. Raman spectra of c-TiO_2 NTs, O-Phos/c-TiO_2 NTs, L-Phe/O-Phos/c-TiO_2 NTs, L-DOPA/L-Phe/O-Phos/c-TiO_2 NTs, and D-DOPA/L-Phe/O-Phos/c-TiO_2 NTs.

Due to the small Raman scattering cross-section of the target chiral molecule, it was difficult to directly employ the characteristic Raman peaks of the DOPA enantiomer for highly sensitive chiral identification.

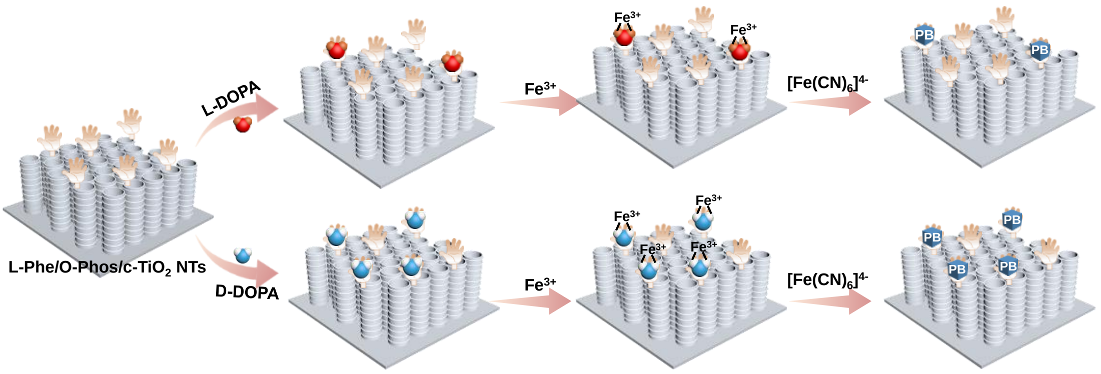

Fig. S22. Schematic illustration of the recognition and quantification of DOPA enantiomers by L-Phe/O-Phos/c-TiO_2 NTs.

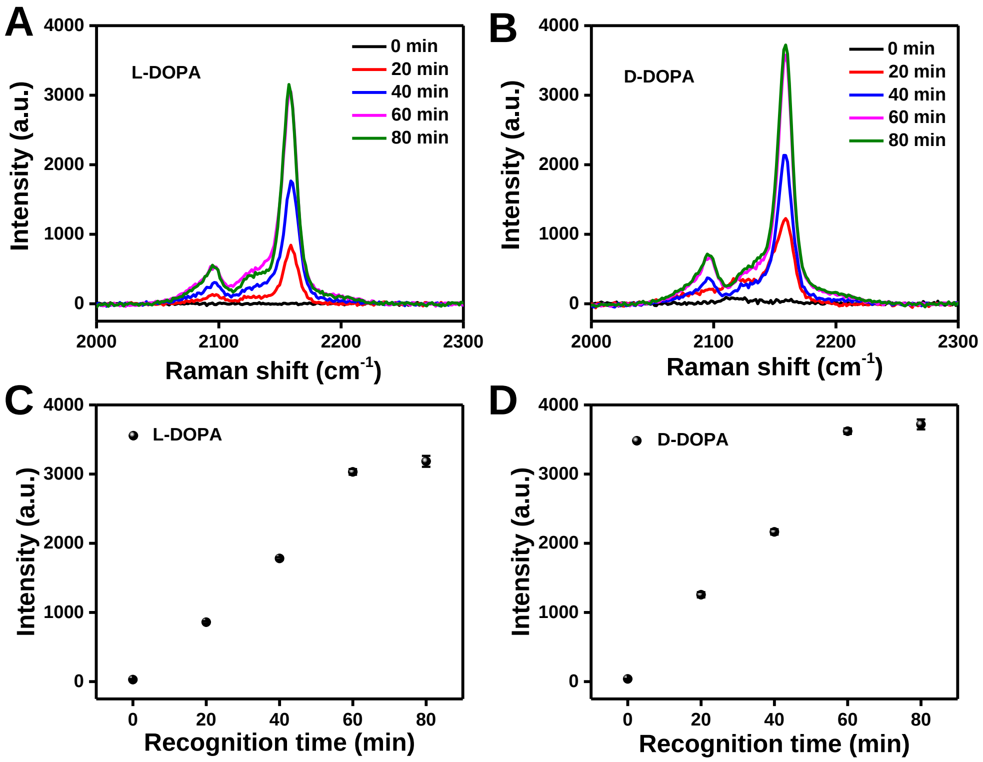

Fig. S23. Raman spectra for sensing (A) L-DOPA and (B) D-DOPA under different recognition time periods. The corresponding Raman intensities for sensing (C) L-DOPA and (D) D-DOPA.

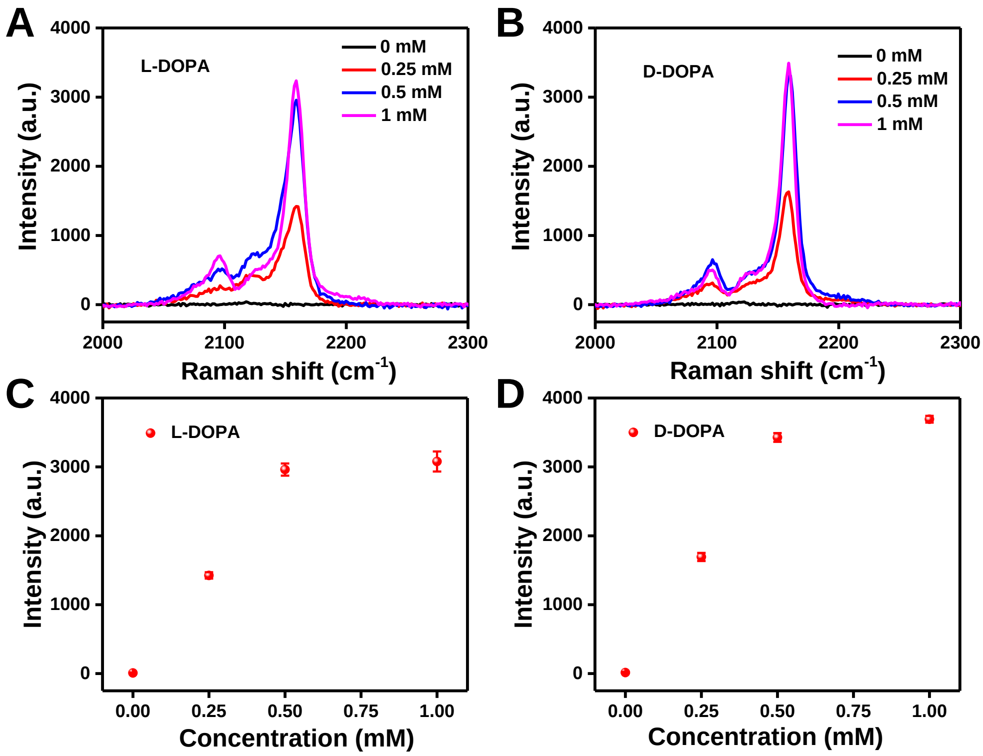

Fig. S24. Raman spectra for sensing (A) L-DOPA and (B) D-DOPA under different Fe^{3+} concentrations. The corresponding Raman intensities for sensing (C) L-DOPA and (D) D-DOPA.

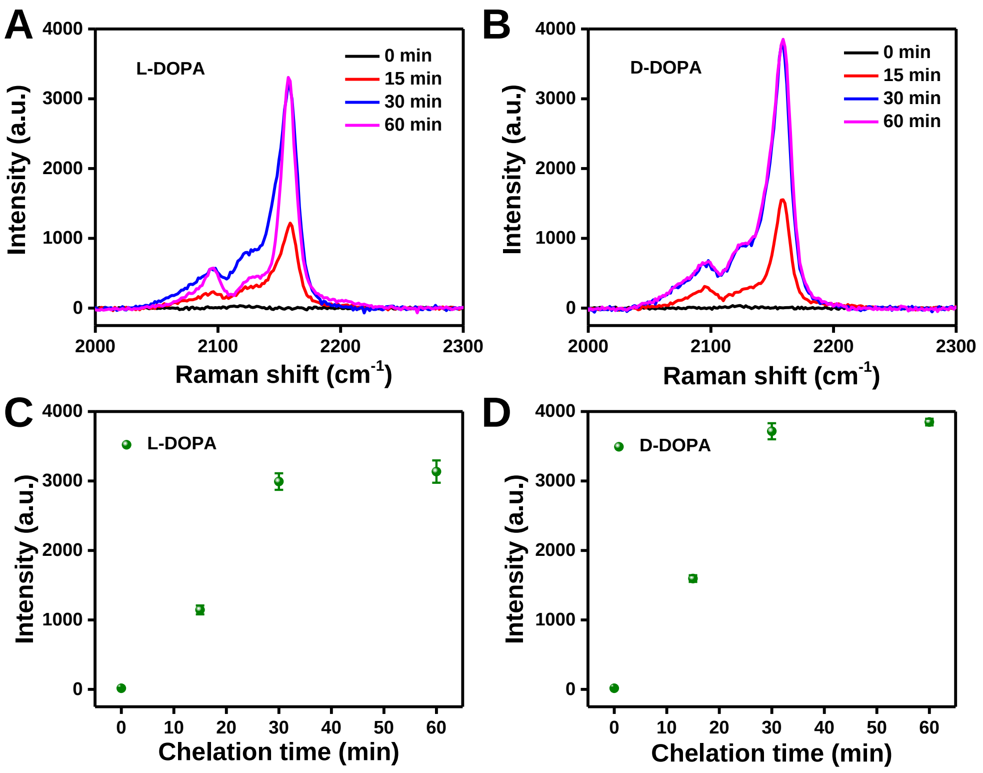

Fig. S25. Raman spectra for sensing (A) L-DOPA and (B) D-DOPA under different Fe^{3+} chelation time periods. The corresponding Raman intensities for sensing (C) L-DOPA and (D) D-DOPA.

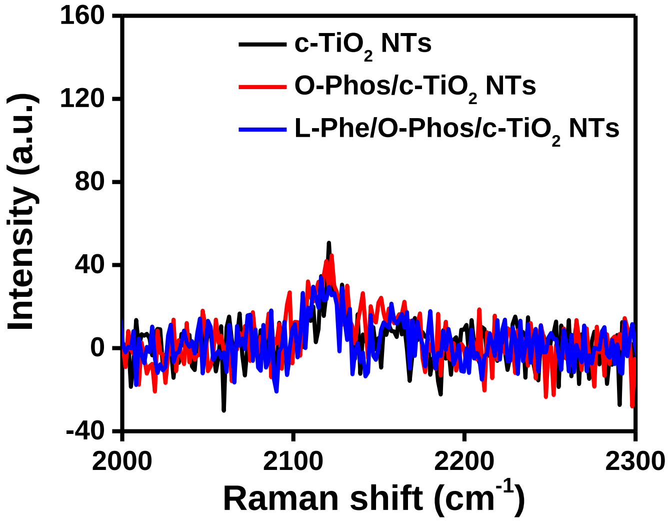

Fig. S26. Raman spectra of c-TiO_2 NTs, O-Phos/c-TiO_2 NTs, and L-Phe/O-Phos/c-TiO_2 NTs after Fe^{3+} and subsequent $[Fe(CN)_6]^{4-}$ incubation in the absence of DOPA enantiomer.

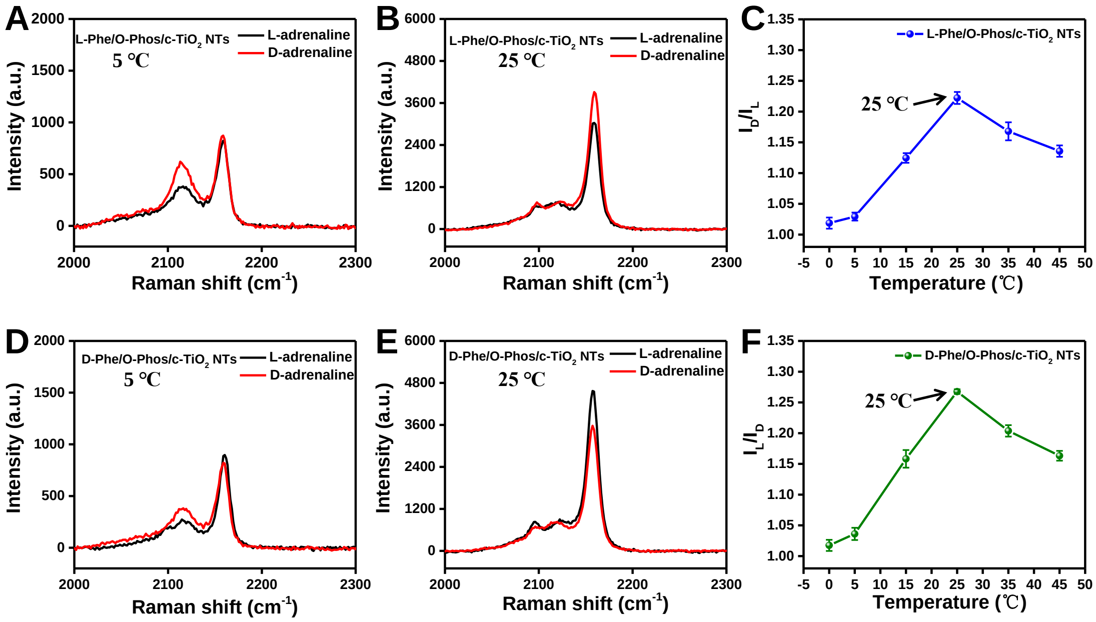

Fig. S27. Raman spectra of PB after L/D-adrenaline recognition by L-Phe/O-Phos/c-TiO_2 NTs at (A) 5 ℃ and (B) 25 ℃. (C) Influence of temperature on the recognition of L/D-adrenaline by L-Phe/O-Phos/c-TiO_2 NTs. Raman spectra of PB after L/D-adrenaline recognition by D-Phe/O-Phos/c-TiO_2 NTs at (D) 5 ℃ and (E) 25 ℃. (F) Influence of temperature on the recognition of L/D-adrenaline by D-Phe/O-Phos/c-TiO_2 NTs.

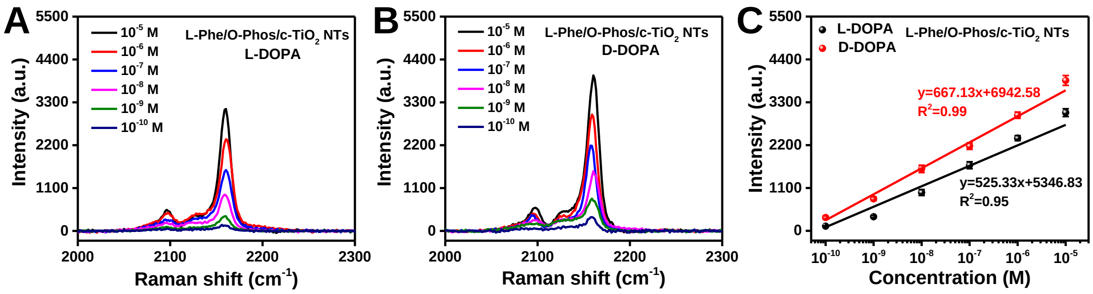

Fig. S28. Raman spectra of PB for sensing different concentrations of (A) L-DOPA and (B) D-DOPA on L-Phe/O-Phos/c-TiO_2 NTs, and (C) the corresponding Raman intensities at 2158 cm^{-1}.

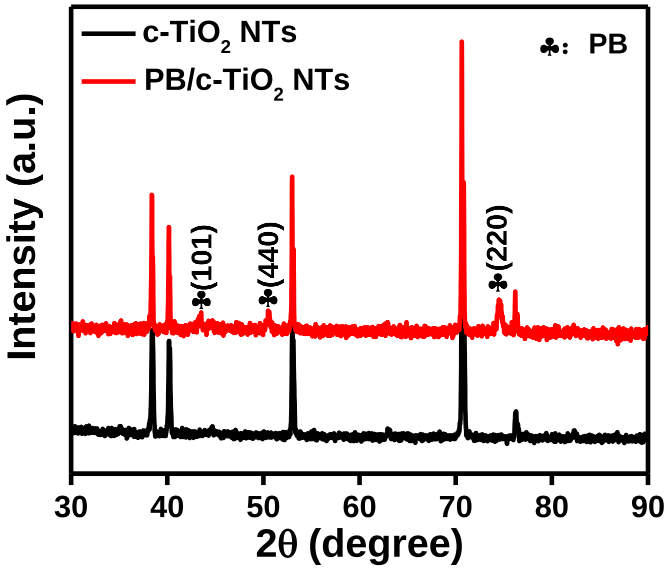

Fig. S29. XRD patterns of c-TiO_2 NTs based SICERS chip before and after applying for D-DOPA sensing and then PB deposition.

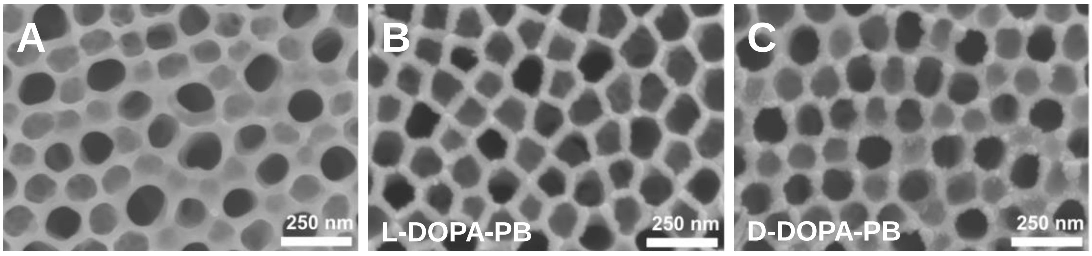

Fig. S30. SEM images of (A) L-Phe/O-Phos/c-TiO_2 NTs (A) before and after recognition of (B) L-DOPA and (C) D-DOPA and then on-site PB deposition.

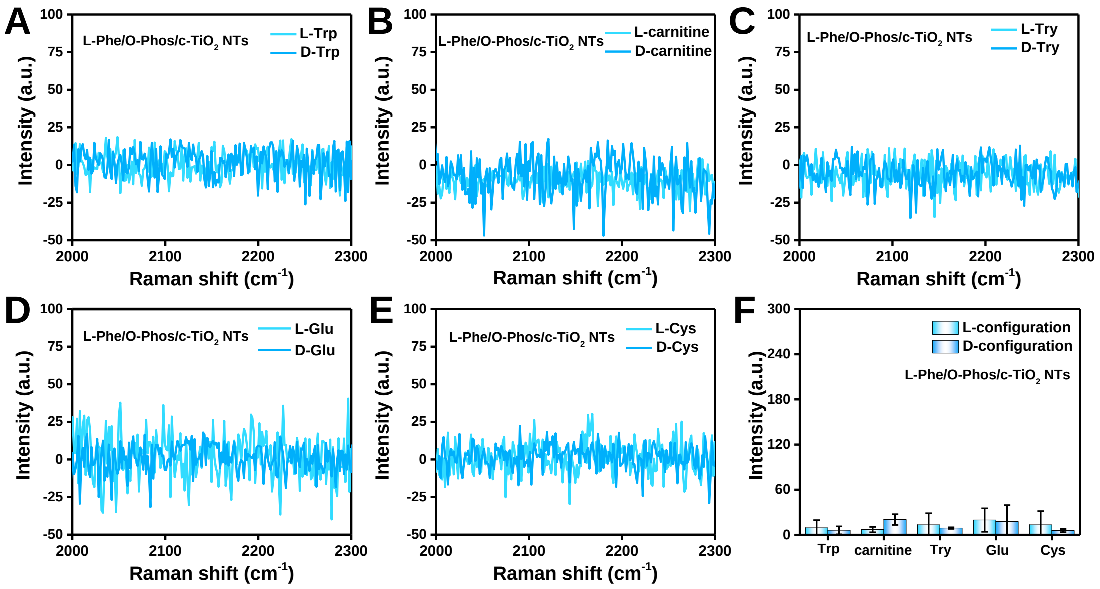

Fig. S31. Raman spectra of PB for recognition of different amino enantiomers on L-Phe/O-Phos/c-TiO_2 NTs: (A) L/D-Trp, (B) L/D-carnitine, (C) L/D-Try, (D) L/D-Glu, (E) L/D-Cys. (F) The corresponding Raman intensity at 2158 cm^{-1}.

REFERENCES

1. J. Xu, H. X. He, X. X. Jian, K. Z. Qu, J. W. Xu, C. W. Li, Z. D. Gao and Y. Y. Song, *Anal. Chem.*, 2021, **93**, 9286−9295.
2. H. Z. Sun, S. Cong, Z. H. Zheng, Z. Wang, Z. G. Chen and Z. G. Zhao, *J. Am. Chem. Soc.*, 2019, **141**, 870−878.
3. J. L. Guo, L. L. Yang, Z. D. Gao, C. X. Zhao, Y. Mei and Y. Y. Song, *ACS Catal.*, 2020, **10**, 5949−5958.
4. K. S. Mun, S. D. Alvarez, W. Y. Choi and M. J. Sailor, *ACS Nano*, 2010, **4**, 2070–2076.
